# Effects of organic fertilizers on yield, soil physico-chemical property, soil microbial community diversity and structure of *Brassica rapa* var. *Chinensis*

**DOI:** 10.3389/fmicb.2023.1132853

**Published:** 2023-05-31

**Authors:** Xia Zhang, Jian Li, Le Shao, Feng Qin, Jie Yang, Hongru Gu, Pin Zhai, Xiaoqing Pan

**Affiliations:** ^1^Institute of Animal Science, Jiangsu Academy of Agricultural Science, Nanjing, China; ^2^Key Laboratory of Crop and Livestock Integrated Farming, Ministry of Agriculture and Rural, Nanjing, China

**Keywords:** *Brassica rapa*, var., *Chinensis*, organic fertilizer, yield and quality, soil property, microbial community

## Abstract

The amount of chemical fertilizer for vegetables is on the high level in China. The use of organic fertilizers to meet the nutrient requirement of crops will be an inevitable practice in sustainable agriculture. In this study, we compared the effects of pig manure fertilizer, rabbit manure fertilizer and chemical fertilizer on yield, quality of *Brassica rapa* var. *Chinensis*, soil physico-chemical properties and microbial community by using two consecutive seasons of three fertilizers in a pot experiment. The results were as follows: (1) In the first season, the fresh yield of *Brassica rapa* var. *Chinensis* applying chemical fertilizer was significantly (*p* ≤ 5%) higher than those of applying the pig manure and rabbit manure fertilizer, and the results were the opposite in the second season. The total soluble sugar concentration of fresh *Brassica rapa* var. *Chinensis* applying rabbit manure fertilizer was significantly (*p* ≤ 5%) higher than those of applying pig manure fertilizer and chemical fertilizer in the first season, and the NO_3_-N content of fresh *Brassica rapa* var. *Chinensis* on the contrary. (2) The organic fertilizer increased the concentration of total nitrogen, total phosphorus and organic carbon in soil in both two seasons. Rabbit manure fertilizer increased the soil pH and EC and significantly (*p* ≤ 5%) reduced the soil NO_3_-N content. (3) The pig manure and rabbit manure fertilizer significantly (*p* ≤ 5%) increased the diversity and abundance of soil bacterial of *Brassica rapa* var. *Chinensis*, but had no significant effect on soil fungi. Pearson correlation analysis showed that soil TN, TP, organic carbon content and EC were significantly correlated with soil bacterial α - diversity. There were significant differences (*p* ≤ 5%) in the bacterial community structures between three treatments in two seasons, and significant differences (*p* ≤ 5%) in the fungal community structures between fertilizer treatments while not between two seasons. Pig manure and rabbit manure fertilizer decreased the relative abundance of soil *Acidobacteria* and *Crenarchaeota*, rabbit manure fertilizer significantly increased the abundance of *Actinobacteria* in the second season. Distance-based redundancy analysis (dbRDA) showed that soil EC, TN, and organic carbon content were key physico-chemical factors in determining bacterial community structure in *Brassica rapa* var. *Chinensis* soil, and soil NO_3_-N, EC, SOC concentration and soil pH in the fungal community structure.

## Introduction

Vegetables are important agricultural products that are essential to the livelihoods of urban and rural residents. China is the world’s largest producer and consumer of vegetables, accounting for more than 40% of the world’s sown area and production(Huang et al., 2017). China’s total fertilizer dose is at a high level, much higher than the United States, the European Union and other developed countries. In particular, the amount of fertilizer applied to vegetables is high, with the average amount of fertilizer applied to vegetables being 445.5 kg/hm^2^ higher than the United States, and 471 kg/hm^2^ higher than the EU (China, T. M. O. A. A. R. A. O. T. P. S. R. O, 2017). According to statistics, the input of N, P_2_O_5_ and K_2_O in the greenhouse vegetables in China is 1.9, 5.4, 1.6 times of the recommended amount respectively, and the amount of chemical fertilizer nutrients was as high as 1,354.5 kg/hm^2^, which is 4.1 times the average amount of the national crop (Huang et al., 2017). In vegetable production, especially in protected vegetable production, the excessive and irrational use of chemical fertilizers not only leads to low fertilizer use and production efficiency, but also causes to a series of serious problems, such as the reduction of soil organic matter content, massive enrichment of available nutrients (nitrogen, phosphorus, etc.), secondary salinization, accumulation of heavy metals, edible parts of vegetables and excessive nitrate in groundwater, which seriously limits the sustainable development of China’s vegetable industry (He et al., 2016; Yang et al., 2016; Wang et al., 2018).

The application of organic fertilizer, which has long been proposed globally, helps to reduce agricultural dependence on chemical fertilizers, in order to prevent soil degradation (Jude and Vidah, 2008; Sun et al., 2019). Organic fertilizers can not only effectively promote the reduction and efficiency of chemical fertilizers (Wang et al., 2020b), and improve crop yield and quality (Zhang et al., 2019; Serri et al., 2021), but also enhance soil microbial diversity, biomass and activity (Gu et al., 2017; Huang et al., 2020), which can subsequently improve soil quality, and contribute to climate protection by increasing carbon sequestration in agricultural ecosystems (Burger and Jackson, 2003; Birkhofer et al., 2008; Zhang et al., 2016). Studies have shown that soil microorganisms are important components of soil ecosystems, and the community composition and diversity of soil microorganisms are important indicators of the ecological function of soil microbial communities (Dong et al., 2014). Organic fertilizers provide a variety of C compounds with different chemical compositions, ranging from readily degradable to stable, which can be utilized by soil microorganisms during mineralization to increase their growth rates and biomass (Lazcano et al., 2021). Thus, organic fertilizers have strong, short- and long-term effects on the soil microbiome and are the basis for supporting soil health by increasing microbial activity, microbial interactions and nutrient cycling (Lazcano et al., 2013; Ling et al., 2016).

With the rapid intensive and large-scale development of livestock production in China, the amount of manure is large and concentrated. According to statistics, the annual production of livestock and poultry manure in China is about 3.8 × 10^9^ t in China (China, M. O. E. A. E. O. T. P. S. R. O., N. B. O. Statistics & M. O. A. A. R. A. O. T. P. S. R. O. China, 2010). By the end of 2019, the comprehensive utilization rate of national livestock and poultry manure reached 75% (China, T. M. O. A. A. R. A. O. T. P. S. R. O, 2017). Thus, the Chinese government attaches great importance to the resource utilization of livestock and poultry manure and encourages the use of organic fertilizers (Dong et al., 2019). Conventional organic fertilizer in China is mainly pig manure and poultry manure. Rabbit farming with regional characteristics is more common only in certain regions. Rabbits are herbivores animals and the crude fiber content of their feed is high. The crude fiber content of domestic rabbit feed is about 13%, and the crude cellulose content of fattening pig feed does not exceed 5%. Compared with pig manure, rabbit manure has a high crude fiber content and can be easily heated up and fermented during composting, and the pH value of both fresh rabbit manure and rabbit manure compost is relatively high. Previous research has shown that the long-term application of chemical fertilizers causes soil acidification and that organic fertilizers can increase soil pH and soil organic carbon and reduce soil acidification (Wang et al., 2019; Liu et al., 2021). Compared to chemical fertilizers, organic fertilizers can substantially increase the organic carbon content of the soil, thus promoting the diversity of soil microorganisms (Wang et al., 2019). In particular, farmers prefer to apply rabbit manure organic fertilizer on acidic soils to improve fruit yield and quality. However, the differences in the effects of chemical fertilizer, rabbit manure and pig manure organic fertilizer on the yield and quality of leafy green vegetables, the physico-chemical properties of the soil, and the microbial community within the soil are not clear.

We hypothesized that chemical fertilizer, rabbit manure organic fertilizer and pig manure organic fertilizer have different effects on soil physico-chemical properties and microbial community, and affect soil microbial community composition through higher pH and higher organic carbon content of organic fertilizer. In this study, we compared the effects of pig manure organic fertilizer, rabbit manure organic fertilizer and inorganic compound fertilizer on yield, quality of *Brassica rapa* var*. Chinensis* and soil physico-chemical properties by using two consecutive seasons of organic fertilizer application in a pot experiment. We also study the effect of different fertilizers on *Brassica rapa* var*. Chinensis* soil microbial community and ecosystem function, investigate soil micro-ecological mechanisms of different fertilizers to improve yield and quality of *Brassica rapa* var*. Chinensis*, to provide a theoretical basis for high-yield vegetable production and resource use of rabbit and pig manure, and to provide basic data support for the application of agricultural information technology between fertilizer, crop and soil interactions.

## Materials and methods

### Site description

The experimental site for this study was located in the greenhouse of the Jiangsu Academy of Agricultural Sciences in Nanjing, China (32°02′N latitude, 118°52′E longitude). The temperature ranged from 6°C to 28°C from 17 March to 16 April, 2020, and from 11°C to 34°C from 18 April to 19 May 2020 in the greenhouse. The experimental soil was Magan soil which was collected from the experimental field around the greenhouse and the previous crop was wheat. The physical and chemical characteristics properties of the soil are shown in Table 1.

**Table 1:** The physical and chemical properties of experimental soil, the pig manure, and the rabbit manure used in the experiment (dry basis).

	Moisture(%)	Organic carbon (g/kg)	N (g/kg)	P_2_O_5_ (g/kg)	K_2_O (g/kg)	pH	EC (ms/cm)
Soil	14.60	5.06	1.41	0.50	14.46	6.98	0.056
Pig manure fertilizer	18.50	179.80	19.07	14.27	24.71	7.03	2.44
Rabbit manure fertilizer	50.04	251.97	27.06	16.45	31.93	9.28	3.98

### Materials

The variety of *Brassica rapa* var*. Chinensis* is heat resistant “605,” which was purchased from the seed store (Nangjing lvlingseeds, Nanjing, China). The two types of organic fertilizers were pig fermentation bed maturing bedding (pig manure fertilizer) and rabbit manure compost (rabbit manure fertilizer), the physical and chemical properties of which are shown in Table 1. The pig manure fertilizer, which was collected from the pig fermentation bed farm of the Luhe Animal Science Base, Jiangsu Academy of Agricultural Sciences, and which was the fermentation product of pig feces and urine, spent mushroom substrate and rice husk, on which pigs had been reared for 1 year. The rabbit dung fertilizer was produced at the organic fertilizer farm of the Luhe Animal Science Base, Jiangsu Academy of Agricultural Sciences, and which was the product of high temperature fermentation of mixed materials mainly consisting mainly of rabbit dung, Chinese medicine residues and cassava residues. The fresh rabbit dung was collected from the experimental rabbit farm of the Luhe Animal Science Base, Jiangsu Academy of Agricultural Sciences. The chemical fertilizer used in the experiment was the complex fertilizer with the ratio of nitrogen, phosphorus pentoxide and potassium oxide of 15–15-15 (Stanley, Linyi, China).

### Experimental design and sample collection

There were three treatments in the pot experiment (1) NPK chemical fertilizer, with an application rate of 300 kg N/hm^2^ (14.12 g/pot, including 2.12 g N/pot, 2.12 g P_2_O_5_/pot and 2.12 g K_2_O/pot), (2) pig manure organic fertilizer, (3) rabbit manure organic fertilizer, with an application rate of 600 kg N/hm^2^ for both pig manure (273 g/pot, including 4.24 g N/pot, 7.14 g P_2_O_5_/pot and 5.50 g K_2_O/pot) and rabbit manure (313 g/pot, including 4.24 g N/pot, 5.79 g P_2_O_5_/pot and 5.00 g K_2_O/pot) to account for differences in nutrient release and soil uptake during crop growth. Four pots were used for each treatment, for a total of 12 pots. The pot used was 40 cm high and 30 cm in diameter at the top. The experimental soil was mixed and sampled and then filled into the pots. All fertilizers were applied once before sowing 10 cm below the soil surface in two seasons. The first *Brassica rapa* var. *Chinensis* crop was sown on 17 March and harvested on 16 April 2020, the second *Brassica rapa var. Chinensis* crop was sown on 18 April and harvested on 19 May 2020. After emergence, 20 seedlings were planted per pot. The experimental samples from all 20 *Brassica rapa* var. *Chinensis* seedlings were collected at the same time as the *Brassica rapa* var. *Chinensis* crop was harvested. After harvesting of *Brassica rapa* var. *Chinensis* crop, 15 cm of soil was removed from the upper pot, mixed well and some of the soil was used for the post-harvest soil samples for each pot.

At harvest, the plants were cut at the soil surface and their fresh weight was determined using a digital balance. After washing the plants with distilled water, each *Brassica rapa* var. *Chinensis* sample was divided into three parts, snap frozen in liquid nitrogen and stored at −18°C for analysis of sugar content, Vc content and NO_3_-N content, respectively. After sieving (< 5 mm) and thorough mixing, each soil sample was divided into two parts: one part was stored at −18°C for the analysis of basic soil properties (pH, EC, NO_3_-N) and for DNA extraction, and the second part was air-dried at room temperature for the measurement of nutrients including nitrogen, phosphorus and organic carbon.

### Fresh *Brassica rapa* var. *Chinensis* quality analysis

#### Total soluble sugar

The total soluble sugar concentration of fresh *Brassica rapa* var. *Chinensis* samples was determined by the anthrone method(Sinay and Karuwal, 2014). Briefly, 0.5–1.0 g of fresh tissue from each *Brassica rapa* var. *Chinensis* sample and 15 mL of distilled water were placed in a stoppered test tube and incubated in a boiling water for 20 min, and then the test tube was cooled at room temperature. The extract solution was filtered into a 50 mL volumetric flask through double circle quantitative filter paper (Grade 2, Ge biotechnology Co., Ltd., Hangzhou, China) (extract twice). The residue of each sample was rinsed with distilled water into the volumetric flask with distilled water and then the volume was fixed on the scale. The diluted sample extract was pipetted 1.0 mL into a 20 mL graduated test tube. 5 mL of anthranilone concentrated sulfuric acid reagent was added one at a time to the test tube and shaken thoroughly, the test tube was immediately placed in a boiling water bath and each tube was kept warm for 10 min. Finally, the absorbance was read (l = 620 nm) in an ultra microplate spectrophotometer (BioTek epoch., Agilent Technologies, Inc., Vermont, USA). The amount of sugar in the extract was calculated using the standard linear equation for glucose and the total sugar content of the test samples was calculated.

#### NO_3_–N in plant

The NO_3_–N concentration was measured using a rapid colourimetric salicylic acid nitration assay (Cataldo et al., 1975). 2.0 g fresh *Brassica raps var. Chinensis* samples, which were cut into pieces and mixed evenly, were added to 10 mL of deionized water in a 20 mL glass test tube. The tube was then placed in a boiling water bath for 30 min, removed and cooled with tap water.

The extract solution was filtered through quantitative filter paper (BioTek epoch, Agilent Technologies, Inc., Vermont, USA) into a 25 mL volumetric flask and the volume was fixed on the balance. Two portions of 0.1 mL of extract were transferred to two test tubes, 0.4 mL of salicylic acid was added to each test tube, mixed well and left at room temperature for 20 min. 9.5 mL of 8% NaOH was added to the test tube, the test tube was shaken well and then cooled to room temperature. Finally, the absorbance (l = 410 nm) was read in an ultra-micro microplate spectrophotometer (BioTek epoch., Agilent Technologies, Inc., Vermont, USA). The amount of NO_3_-N in the extract was calculated from the standard linear equation for KNO_3_ and the plant NO_3_-N content in the test samples was calculated.

### Soil physical and chemical analysis

Total nitrogen (TN) was determined by the Kjeldahl method using a Kjeldahl Nitrogen Detector (Kjeltec 8,400, FOSS Ltd., Denmark). Total phosphorus (TP) was determined using the method for the determination of total phosphorus in soil (GB9837-88). Total organic carbon (TOC) was determined using a TOC analyzer (Multi N/C 3100, Elementar Analysensysteme GmbH, Germany). Soil pH was determined using the potentiometric method (water: soil = 2.5: 1) with a pH meter (FiveEasy Plus pH/mV, Mettler-Toledo (Schweiz) GmbH, Switzerland). Soil EC (soil electrical conductivity, water: soil = 10: 1) was measured with a conductivity meter (EC215 Conductivity Meter, Hanna Instruments, Italy).

NO_3_–N in soil is determined by dual-wavelength ultraviolet spectrophotometry (Norman et al., 1985). Briefly, 10 g of the soil samples, which were frozen at – 18°C, 100 mL of 2 M KCL (1:5 soil to solution ratio) and 1 g of non-phosphate activated carbon powder were added to an Erlenmeyer flask, and shaken in a constant temperature (refrigerated) oscillator (Taicang Huamei instrument factory, Taicang, China) for 1 h. The extract solution was filtered through quantitative filter paper (BioTek epoch, Agilent Technologies, Inc., Vermont, USA) into a 50 mL plastic reagent bottle. And then the soil NO_3_–N concentration was measured by ultraviolet spectrophotometry using an ultra micro microplate spectrophotometer (BioTek epoch., Agilent Technologies, Inc., Vermont, USA) (GB/T32737-2016).

Differences in yield, *Brassica rapa* var. *Chinensis* quality and soil physicochemical characteristics between fertilizer treatments were determined using one-way analysis of variance (ANOVA). The Pearson’s coefficients were used to correlate yield, *Brassica rapa* var. *Chinensis* quality and soil physicochemical characteristics.

### 16S rRNA and ITS1-5f sequencing of the soil

Total genomic DNA was extracted from 200 mg soil samples using the CTAB method. The concentration and purity of the extracted DNA was monitored on 1% agarose gels. The DNA was then stored at – 80°C until further processing. The v4 region of the bacterial 16S rRNA gene was amplified using the common primer pair F (5′-CCTAYGGGRBGCASCAG-3′) and R (5′-GGACTACNNGGGTATCTAAT - 3′) with the barcode. The ITS1 - 5f of the fungal gene was amplified with the common primer pair F (G5’ - GGAAGTAAAAGTCGTAACAAGG - 3’) and R (5′-GCTGCGTTCTTCATCGATGC-3′) with the barcode. PCR reactions were performed using 15 μl Phusion® High-Fidelity PCR Master Mix (New England Biolabs); 0.2 μM forward and reverse primers and approximately 10 ng template DNA. Thermal cycling conditions were as follows: an initial denaturation at 98°C for 1 min, followed by 30 cycles at 98°C for 10 s, 50°C for 30 s and 72°C for 30 s, with a final extension at 72°C for 5 min. Finally, an equal volume of 1X loading buffer (containing SYB green) was mixed with all PCR products and electrophoresed on a 2% agarose gel for detection. PCR products were mixed at equidensity. The mixed PCR products were then purified using Qiagen Gel Extraction Kit (Qiagen, Germany). Sequencing libraries were generated using a TruSeq DNA PCR-Free Sample Preparation Kit (Illumina, USA) according to the manufacturer’s recommendations and index codes were added. Library quality was assessed using the Qubit@ 2.0 Fluorometer (Thermo Scientific) and the Agilent Bioanalyzer 2,100 system. Finally, the library was sequenced on an Illumina NovaSeq platform and 250 bp paired-end reads were generated.

### Data analysis 16S rRNA and ITS1-5f sequencing of the soil samples

Mitochondria and chloroplast sequences were removed prior to analysis. Paired-end reads were assigned to samples based on their unique barcode and truncated by cutting off the barcode and primer sequence. Paired-end reads were merged using FLASH (Magoč and Salzberg, 2011). Quality filtering of the raw tags was performed under specific filtering conditions (Q score for quality trimming = 19, minimum length = 3) to obtain the high-quality clean tags (Bokulich et al., 2013) according to the quality controlled process of Quantitative Insights Into Microbial Ecology (QIIME) (Caporaso et al., 2010). The tags were compared with the reference database (Silva database, https://www.arb-silva.de/) using the UCHIME algorithm (Edgar et al., 2011) to detect chimera sequences, and then the chimera sequences were removed (Haas et al., 2011). Finally, the effective Tags were obtained.

Sequence analysis was performed using Uparse software (Edgar, 2013) and sequences with ≥97% similarity were assigned to the same OTUs with the most abundant selected as the representative sequence for further annotation. For each representative sequence, the Silva database (Quast et al., 2012) was used based on the Mothur algorithm to annotate taxonomic information based on naive Bayesian classification for bacteria and Blast for fungi. Multiple sequence alignments were performed using the MUSCLE software (Edgar, 2004). OTU abundance information was normalized using a standard of sequence number corresponding to the sample with the fewest sequences, which was 55,582 for bacteria and 29,042 for fungi.

Alpha diversity is used to analyze the complexity of species diversity for a sample through 3 indices, including observed-species, Chao1, and Shannon. The Tukey test was used to analyze the differences between groups of the alpha diversity index. All these indices in our samples were calculated by QIIME (version 1.7.0) and displayed by R software (version 2.15.3). Beta diversity NMDS (Bray-Curits distance) was calculated by QIIME software (version 1.9.1) to evaluate the changes in microbial community structure. Based on the Unifrac distance, the amova function of the Mothur algorithm was used to analyze the differences between fertilizer treatment groups. In order to better reflect the non-linear structure of the ecological data, we performed the Non-Metric Multi-Dimensional Scaling (NMDS) of the nonlinear model based on Bray-Curtis distance. Pearson correlation approaches were used to correlate alpha-diversity with physicochemical characteristics of soils. Distance-based redundancy analysis (db-RDA) was then performed to further investigate the influence of soil physiochemical properties on bacteria and fungi according to relative phylum abundance R (version 3.2.2).

## Results

### Effects of different fertilizers on yield and quality of *Brassica rapa* var. *Chinensis*

Table 2 shows the effect of different fertilizers on the yield and quality of *Brassica rapa* var. *Chinensis*. The fresh yield of *Brassica rapa* var. *Chinensis* in the first season were lower than that in the second season. In the first season, the fresh yield of *Brassica rapa* var. *Chinensis* (222.7 g/pot) with chemical fertilizer was significantly (*p* ≤ 5%) higher than that with pig manure and rabbit manure fertilizer, which were 202.3 g/pot and 136.7 g/pot, respectively. And the fresh yield of *Brassica rapa* var. *Chinensis* (638.7 g/pot) with pig manure fertilizer was significantly (*p* ≤ 5%) higher than that with rabbit manure and chemical fertilizer, which were 560.8 g/pot and 537.0 g/pot respectively, in the second season.

**Table 2:** Effect of different fertilizers on the yield and the quality of *Brassica rapa* var. *Chinensis.*

	Planting seasons	Chemical fertilizer	Pig manure fertilizer	Rabbit manure fertilizer
Fresh yield (g/pot)	The first season	222.7 ± 25.c	202.3 ± 19.6c	136.7 ± 19.3d
	The second season	537.0 ± 52.8b	638.7 ± 43.5a	560.8 ± 14.0b
Total soluble sugar (mg/g)	The first season	10.97 ± 3.11b	14.16 ± 3.24b	21.07 ± 1.66a
	The second season	5.48 ± 0.41c	6.27 ± 0.57c	5.63 ± 0.17c
NO_3_-N in plant (mg/kg)	The first season	530.4 ± 107.5c	372.1 ± 61.8c	86.5 ± 49.9d
	The second season	641.4 ± 146.3b	811.7 ± 98.9a	451.8 ± 36.8c

Different lower case letters in each row indicate a significant difference at the 5% level.

The total soluble sugar concentration of *Brassica rapa* var. *Chinensis* in the first season, which ranged from 10.97 mg/g to 21.07 mg/g, was higher significantly than that in the second season, which ranged from 5.48 mg/g to 6.27 mg/g. The total soluble sugar concentration of fresh *Brassica rapa* var. *Chinensis* using rabbit manure fertilizer (21.07 mg/g) was significantly higher (*p ≤ 5%*) than those using pig manure fertilizer and chemical fertilizer, which were 14.16 mg/g and 10.97 mg/g, respectively, in the first season, but there was no significant difference in the second season.

The NO_3_-N content of fresh *Brassica rapa* var. *Chinensis* in the second season was significantly higher than those in the first season (Table 2). The NO_3_-N content of fresh *Brassica rapa* var. *Chinensis* fertilized with chemical fertilizer (530.4 mg/kg) was significantly higher (*p ≤ 5%*) than those fertilized with pig manure and rabbit manure which were 372.1 mg/kg and 86.5 mg/kg, respectively, in the first season, that fertilized with pig manure fertilizer (811.7 mg/kg) was significantly higher (*p ≤ 5%*) than those fertilized with chemical fertilizer and rabbit manure fertilizer which were 645.4 mg/kg and 451.8 mg/kg, respectively, in the second season.

### Effects of different fertilizers on soil nutrients, pH, and EC

Table 3 shows the effect of the different fertilizers on the physical and chemical properties of the soil. Total nitrogen, total phosphorus and organic carbon in the soil were higher in the second season than in the first. In two seasons, soils treated with pig and rabbit manure fertilizers had significantly higher concentrations of total nitrogen, total phosphorus and organic carbon than those treated with chemical fertilizer. However, in the same season, there was no significant difference in total nitrogen, total phosphorus and organic carbon content in the soil after application of pig and rabbit manure fertilizer.

**Table 3:** Effect of different fertilizers on soil nutrients, pH, and EC after crop harvest.

	Planting seasons	Chemical fertilizer	Pig manure fertilizer	Rabbit manure fertilizer
TN (g/kg)	The first season	1.09 ± 0.16c	1.91 ± 0.08b	1.90 ± 0.29b
	The second season	1.75 ± 0.19b	2.67 ± 0.53a	2.71 ± 0.35a
TP (g/kg)	The first season	3.94 ± 0.19d	7.60 ± 0.30c	6.95 ± 0.77c
	The second season	3.78 ± 0.30b	12.11 ± 3.14a	9.72 ± 0.55a
SOC (g/kg)	The first season	6.26 ± 0.56c	16.32 ± 0.91b	15.62 ± 1.20b
	The second season	5.65 ± 0.40c	24.22 ± 0.14a	24.31 ± 1.01a
pH	The first season	6.63 ± 0.05ab	6.41 ± 0.06c	6.60 ± 0.06ab
	The second season	6.44 ± 0.03c	6.62 ± 0.06ab	6.65 ± 0.08a
EC (μs/cm)	The first season	436 ± 38d	423 ± 57d	511 ± 63c
	The second season	691 ± 3a	625 ± 33b	749 ± 19a
NO_3_-N in soil (mg/kg)	The first season	66.01 ± 15.27c	64.38 ± 25.63c	28.74 ± 5.38d
	The second season	107.56 ± 4.33b	146.06 ± 21.50a	65.18 ± 13.47c
C/N	The first season	5.88 ± 1.24b	8.56 ± 0.72a	8.35 ± 1.22a
	The second season	3.38 ± 0.26c	9.06 ± 1.84a	9.04 ± 0.91a

TN: total nitrogen, TP: total phosphorus, SOC: soil organic carbon. Significant differences at the 5% level are indicated by different small letters in each row.

The rabbit and pig manure fertilizer increased the soil pH value from 6.60 and 6.41 at the end of the first season to 6.65 and 6.62 at the end of the second season, while the chemical fertilizer reduced the soil pH value from 6.63 at the end of the first season to 6.44 at the end of the second season. However, compared to the initial soil pH of 6.98 (Table 1), all fertilization treatments reduced the soil pH.

The soil ECs in the second season were significantly higher (*p* ≤ 5%) than those in the first season, and the order of the soil ECs order of the three fertilizers was: rabbit manure fertilizer (511 and 749 μs/cm) > chemical fertilizer (436 and 691 μs/cm) > pig manure fertilizer (423 and 625 μs/cm).

The soil NO_3_-N content in the second season, which ranged from 28.74 to 66.01 mg/kg, was significantly (*p* ≤ 5%) higher than that in the first season, which ranged from 65.18 to 146.06 mg/kg. Pig manure fertilizer and chemical fertilizer significantly increased soil NO_3_-N content compared to rabbit manure fertilizer. The C/N ratio in soil using pig manure and rabbit manure fertilizer, which increased over time, was significantly higher than that with chemical fertilizer, which decreased over time.

Table 4 shows that, there was a significant positive correlation between *Brassica rapa* var. *Chinensis* yield and soil EC, TN, TP, and nitrate, a significant negative correlation between *Brassica rapa* var. *Chinensis* total soluble sugar and soil EC and nitrate content, and a significant positive correlation between *Brassica rapa* var. *Chinensis* nitrate content and soil nitrate content.

**Table 4:** Pearson’s correlation between yield and quality of *Brassica rapa* var. *Chinensis* and soil physico-chemical properties.

	pH	EC	TN	TP	SOC	NO_3_-N in soil	C/N
yield	0.176	0.812**	0.600**	0.473*	0.421*	0.731**	0.065
Soluble sugar	−0.117	−0.610**	−0.298	−0.179	−0.151	−0.679**	0.129
Nitrate of *Brassica Chinensis*	−0.038	0.358	0.180	0.188	0.063	0.876**	−0.114

* and ** indicate significance (*p* ≤ 0.05) and high significance (*p* ≤ 0.01).

### Sequence data and richness and α-diversity of bacterial and fungal community

**Figure 1:**
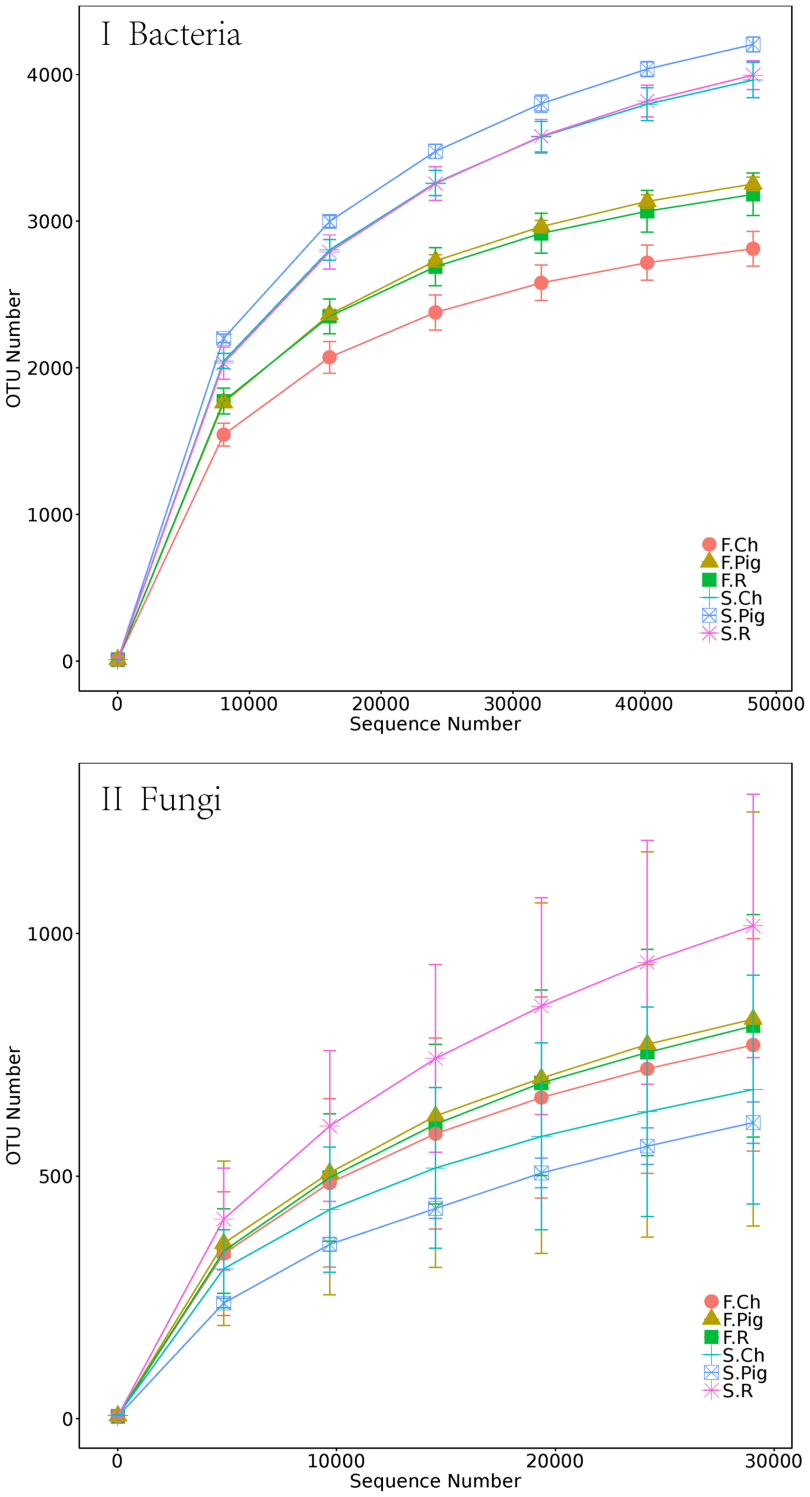
The rarefaction curves of bacterial and fungal rRNA sequencing depth and number of species number in *Brassica rapa* var. *Chinensis* soil.

After the quality control, 1,477,222 and 1,556,272 valid tags were obtained from 16S rRNA and ITS rRNA gene sequencing. After 97% OTU clustering, 10,613 bacterial OTUs and 4,928 fungal OTUs were retained. Rarefaction curves showed that the sequencing effort was sufficient to describe the majority of the diversity in *Brassica rapa* var. *Chinensis* soil samples (Figure 1).

The observed OTUs, Chao1 and Shannon indices (Table 5) were evaluated to estimate the alpha diversity of bacterial and fungal communities in *Brassica rapa* var. *Chinensis* soils under different fertilized treatments. According to Table 5, we found that the soil sample of each fertilizer treatment in the second season had the higher observed OTUs, Chao1 index and Shannon index of bacterial community than that in the first season, and there are significant difference between treatments. The observed OTUs, Chao1 index and Shannon index of pig and rabbit manure treatments were significantly higher than that of chemical fertilization treatment in the same season. These results indicated that the soil samples of pig manure and rabbit manure fertilizer had the higher level of bacterial diversity than that of chemical fertilizer in both two seasons. We found that the Observed OTUs, Chao1 index and Shannon index of fungal community of the soil sample of three fertilizer treatments in two seasons had no significant difference due to the large variance within treatments, but the Chao1 of organic fertilization treatment, especially rabbit manure fertilization treatment, was higher than that of chemical fertilizer treatment.

**Table 5:** The Chao1 index and Shannon index of bacterial and fungal community.

Planting seasons	Treatments	Bacteria	Fungi
		Observed OTUs	Chao 1	Shannon	Observed OTUs	Chao 1	Shannon
The first season	Ch	3,085 ± 131d	3,423 ± 119d	8.90 ± 0.34c	766 ± 249a	1,012 ± 231a	4.17 ± 1.72a
	Pig	3,463 ± 167c	3,791 ± 136c	9.60 ± 0.19b	832 ± 493a	1,072 ± 617a	4.30 ± 0.94a
	R	3,578 ± 66c	3,943 ± 91c	9.58 ± 0.06b	818 ± 270a	1,098 ± 356a	4.10 ± 0.92a
The second season	Ch	4,011 ± 111b	4,397 ± 136b	9.48 ± 0.37b	675 ± 233a	915 ± 346a	4.71 ± 0.51a
	Pig	4,332 ± 89a	4,741 ± 90a	10.36 ± 0.04a	621 ± 39a	973 ± 49a	3.40 ± 0.39a
	R	4,210 ± 171a	4,658 ± 167a	10.03 ± 0.40a	1,006 ± 310a	1,409 ± 474a	4.52 ± 0.75a

Significant differences at the 5% level are indicated by different lower case letters in each column.

### β-Diversity of bacteria and fungi under three fertilization treatments

**Figure 2:**
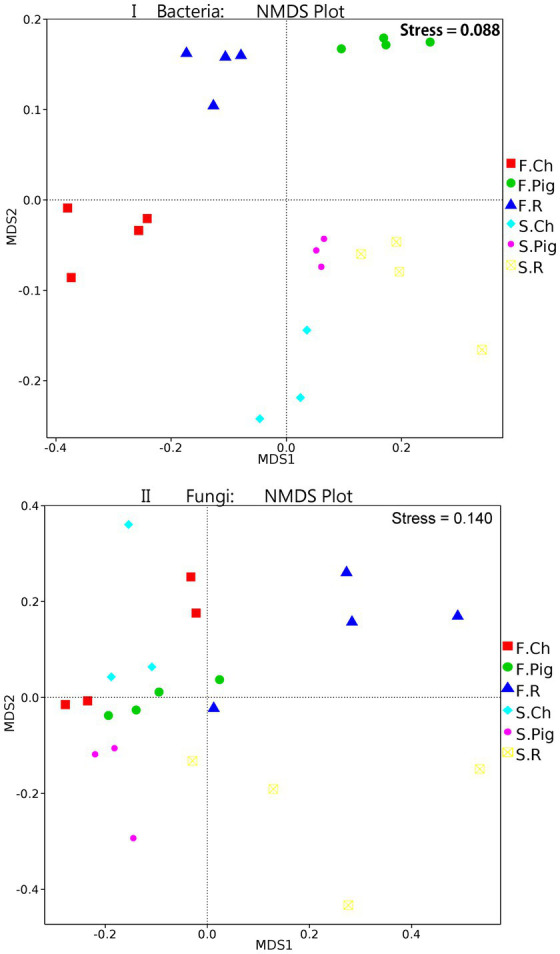
NMDS (Bray–Curits distance) of bacterial and fungal communities under three fertilization treatments in two seasons.

Analysis of molecular variance (AMOVA) showed that the differences in beta diversity were significant (bacteria: Fs = 6.76, *p* ≤ 0.001*; fungi: Fs = 2.40, *p* ≤ 0.001*) between the fertilizer treatment groups. The beta diversity (NMDS) of soil microbial communities of *Brassica rapa* var. *Chinensis* under fertilizer treatments was further investigated. The non-metric multi-dimensional scaling (NMDS) plot (Figure 2I) of the bacterial communities showed a clear separation among the soil bacterial of *Brassica rapa* var. *Chinensis* under two seasons and three fertilizer treatments, the bacterial communities were distinguished along MDS1 in two seasons and along MDS2 for different fertilizer treatments. These results indicated that there were significant differences (*p* ≤ 5%) in the bacterial community structure between three treatments in two seasons. And the distance between the three fertilizations in the second season was relatively close, indicating that the bacterial community of the three fertilizations in the second season was relatively similar. Thus, pig and rabbit manure fertilization had altered the structure of the soil bacterial community, and the difference in bacterial community structure between the three fertilizer treatments in the first season was significant compared to that in the second season. From the NMDS plot of fungal communities (Figure 2II), there was also a clear distinction between MDS1 and MDS2 of six groups of three fertilizer treatments in two seasons, the fungal communities were distinguished along MDS2 for three fertilizer treatments and not along MDS1 in two seasons. This result was consistent with the Amova analysis which showed that beta diversity was a significant difference in fungal community structure between fertilizer treatments.

### Relative phylum and genus abundance of soil microbial communities

#### Relative phylum and genus abundance of soil bacterial communities

**Figure 3:**
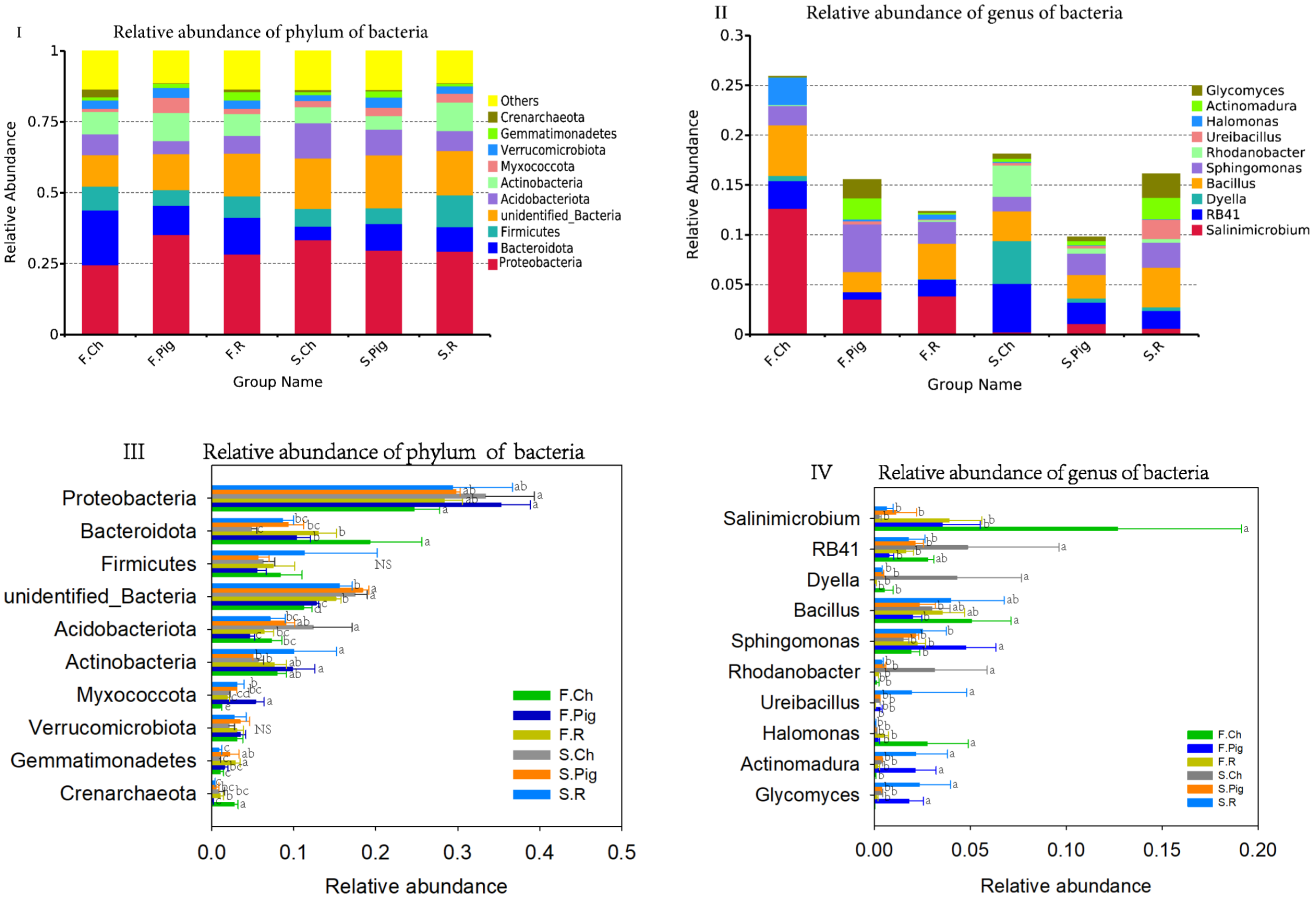
The relative abundance of phylum and genus of soil bacterial communities. Values are the mean of the three or four replicates of each treatment. Vertical bars represent the standard error, and bars with different letters within the same treatment indicate that there are significant differences between treatments at *p ≤* 0.05. NS, not significant.

All bacterial OTUs were classified into 904 genera, 534 families and 78 phyla. The dominant phyla (relative sequence abundance ≥1%) across all samples were *Proteobacteria* (29.99%), *Bacteroidota* (11.25%), and *Firmicutes* (7.55%), *Unidentified_Bacteria* (14.86%), *Acidobacteriota* (7.55%), *Actinobacteria* (7.88%), *Myxococcota* (2.73%), *Verrucomicrobiota* (1.36%), *Verrucomicrobia* (3.00%) and *Gemmatimonadetes* (1.57%), representing 86.37% of the bacterial sequences (Figure 3I). The abundance of *Acidobacteriota* and *Unidentified_Bacteria* in the soil of the second season was significantly higher than that of the first season, the abundance of *Bacteroidota* and *Crenarchaeota* of the second season was significantly lower than that of the first season. Organic fertilization significantly decreased the abundance of soil *Acidobacteriota* and *Crenarchaeota*, increased the abundance of soil *Unidentified Bacteria*, *Myxococcota* and *Gemmatimonadetes*, and rabbit manure fertilization significantly increased the abundance of soil *Actinobacteria* (Figure 3III).

Four dominant genera (relative sequence abundance ≥1%) in all samples were *Salinimicrobium* (3.96%) and *Sphingomonas* (2.57%), which belong to *Proteobacteria*, and RB41 (2.22%) belongs to *Acidobacteriota*, *Bacillus* (3.38%) belong to *Firmicutes,* accounting for 12.12% of the bacterial sequences (Figure 3II). Among the top 10 dominant genura, the abundance of *Dyella* and *Rhodanobacter* in the soil of the second season was significantly higher than that of the first season, the abundance of *Salinimicrobium* and *Sphingomonas* in the second season was significantly lower than that of the first season. Pig and rabbit manure fertilizer significantly increased the abundance of *Sphingomonas*, *Actinomadura* and *Glycomyces* and decreased the abundance of *Salinimicrobium*, *RB41*, *Dyella*, *Rhodanobacter* and *Halomonas* (Figure 3IV).

#### Relative phylum and genus abundance of soil fungal communities

**Figure 4:**
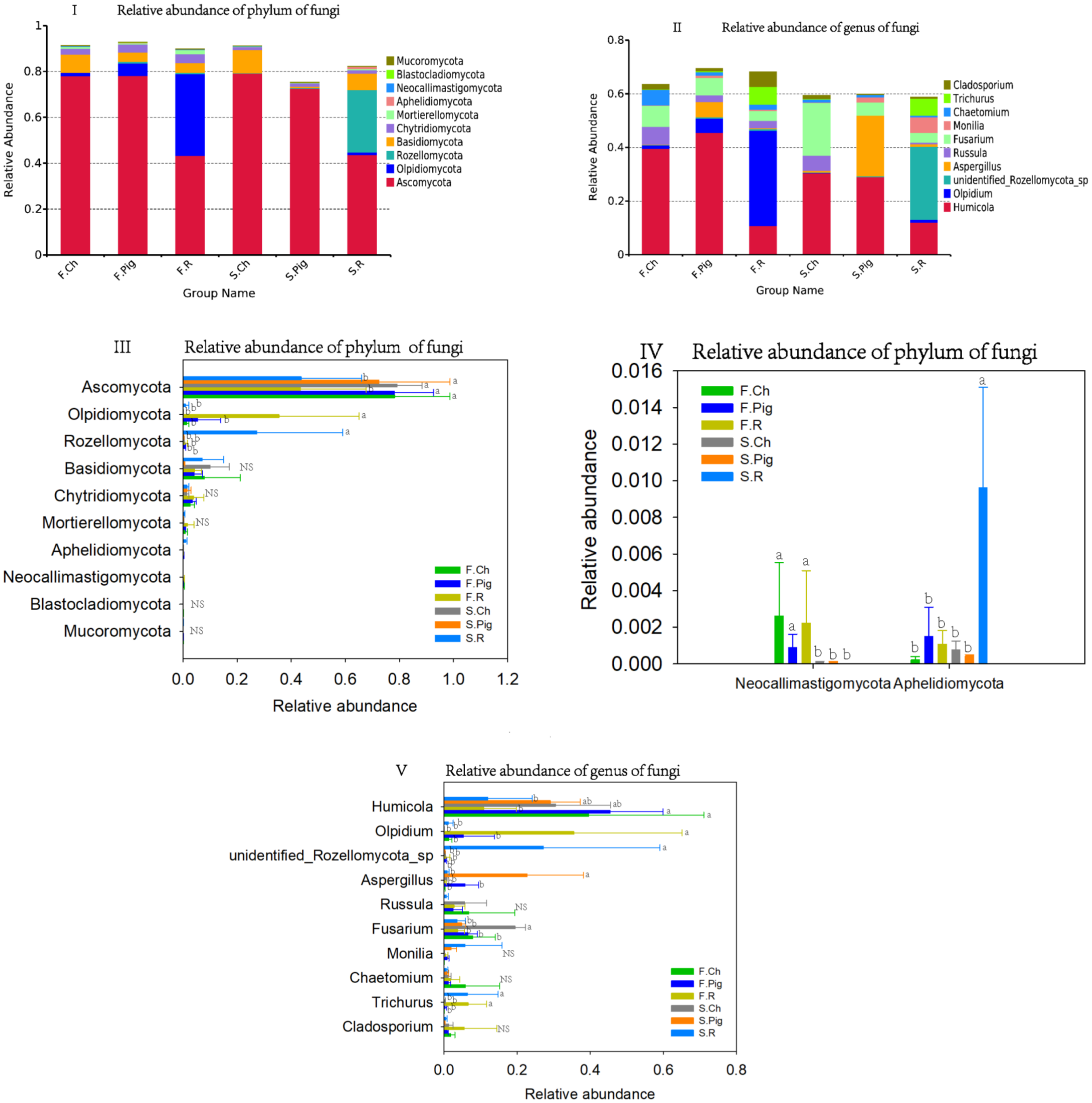
The relative abundance of the phylum and genus of the soil fungal communities. Values are the mean of the three or four replicates of each treatment. Vertical bars represent the standard error, and bars with different letters within the same treatment indicate that there are significant differences between treatments at *p ≤* 0.05. NS, not significant.

All fungal OTUs were classified into 743 genera, 743 families and 18 phyla. The dominant phyla (relative sequence abundance ≥1%) in all samples were *Ascomycota* (64.95%), *Olpidiomycota* (7.90%), *Basidiomycota* (5.66%), *Rozellomycota* (5.27%), *Chytridiomycota* (2.47%), accounting for 85.96% of the fungal sequences (Figure 4I). The abundance of Ascomycota in the soil of the chemical and pig manure fertilization treatments was significantly higher than that of the rabbit manure fertilization treatment in both two seasons. The abundance of *Olpidiomycota* in the soil of rabbit manure fertilization treatments was significantly higher than that of the chemical and pig manure fertilization treatments in both two seasons. The abundance of *Rozellomycota* in the soil of the rabbit manure fertilization treatment was significantly higher than that of the other treatments in the second season.

Chemical fertilizer increased the abundance of soil *Basidiomycota* in the soil, and the abundance of *Chytridiomycota* was higher in the first season than in the second season (Figures 4III,IV).

The six dominant genera (relative sequence abundance ≥1%) in all samples were *Humicola* (27.74%), *Olpidium* (7.89%), *Fusarium* (7.27%), *unidentified Rozellomycota* sp. (5.20%), *Aspergillus* (4.49%), *Russula* (3.08%), *Trichurus* (2.47%), *Chaetomium* (2.04%), *Cladosporium* (1.82%) and *Monilia* (1.58%), accounting for 63.57% of the fungal sequences (Figure 4II). Among the top 10 dominant genera, rabbit manure fertilizer significantly reduced the abundance of *Humicola*, and increased the abundance of *Olpidium* and *Trichurus*. Pig manure fertilizer significantly increased the abundance of Aspergillus. Pig and rabbit manure fertilizer reduced the abundance of *Fusarium* (Figure 4V).

### Soil microbial community structures and their relationships with soil properties

#### Pearson’s correlation between soil physico-chemical properties and microbial community alpha diversity

We estimated the Pearson correlation between soil physicochemical properties and microbial community alpha diversity measures (Table 6). We found that EC, TN, TP and SOC were significantly positively correlated (*p* ≤ 1%) with bacterial Chao 1, and negatively correlated with Goods coverage. TN, TP, and SOC concentrations were a significantly positively correlated (*p* ≤ 1%) with Shannon of the bacterial communities. Therefore, the EC, TN, TP and SOC concentrations were significantly correlated with alpha diversity in soil bacterial communities under three fertilizer treatments. However, the results showed that pH, EC, TN, TP, SOC, NO_3_-N and C/N had no significant correlations with the observed OTUs, Shannon, chao1, of the fungal communities.

**Table 6:** Pearson’s correlation between soil physicochemical properties and alpha-diversity of soil microbial communities of *Brassica rapa* var. *Chinensis*.

	Observed OTUs	Shannon	Chao1	Observed OTUs	Shannon	Chao1
	Bacteria			Fungi		
pH	0.175	0.201	0.217	0.279	0.024	0.232
EC	0.759**	0.417	0.799**	0.145	0.222	0.193
TN	0.748**	0.747**	0.781**	0.046	−0.040	0.067
TP	0.696**	0.794**	0.720**	0.019	−0.124	0.021
SOC	0.635**	0.741**	0.659**	0.185	−0.178	0.202
NO_3_-N	0.425*	0.205	0.392	−0.174	0.032	−0.187
C/N	0.317*	0.468**	0.334	0.180	−0.378	0.233

TN, total nitrogen; TP, total phosphorus; SOC, soil organic carbon. df = 21, *p*_0.05_ = 0.413, *p*_0.01_ = 0.526.

### Distance-based redundancy analysis (dbRDA) of soil physico-chemical properties and microbial community

**Figure 5:**
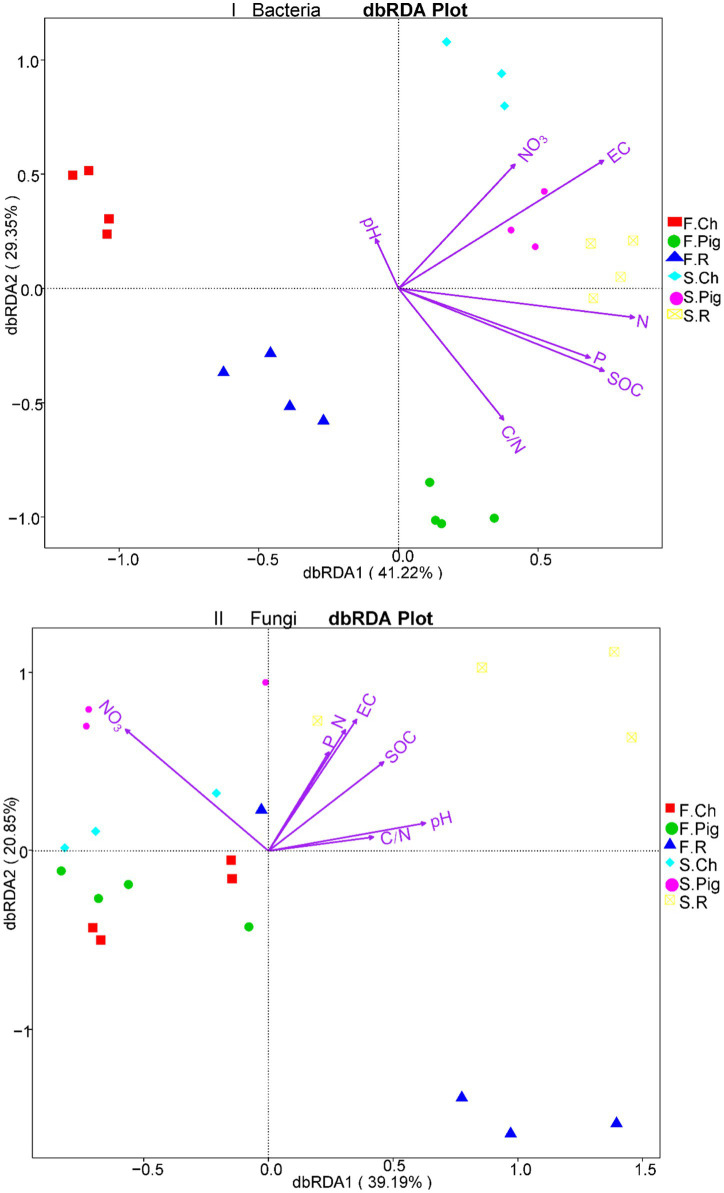
db-RDA plot of microbial communities under three fertilization treatments.

To investigate the effect of the three fertilizer treatments on the composition of the bacterial and fungal communities and environmental factors, and to further reveal the relationship between soil physicochemical properties and microbial community structures, a distance-based redundancy analysis (db-RDA) of soil physicochemical properties and the relative abundance of OTUs of the microbial community was performed (Figure 5). The dbRDA1 and dbRDA2 explained 70.57% of the diversity of the bacterial community and 60.04% of the diversity of the fungal community. The bacterial community was clearly separated among the three fertilizer treatments in two seasons, and the fungal community was not clearly separated (Figure 5). The db-RDA plot (Figure 5I) clearly showed that soil EC, TN, and SOC concentrations were the three longest vectors, and they could be key physicochemical factors to assemble the bacterial community structure in the experimental soil. In the second season, the soil bacterial community of pig manure fertilization treatment was positively correlated with soil EC, that of rabbit manure fertilization treatment was positively correlated with soil TN content, and that of the chemical fertilization treatment was positively correlated with soil NO_3_^−^N content. The db-RDA plot (Figure 5II) clearly showed that soil NO_3_-N, EC, SOC concentration and pH were the four longest vectors. The soil fungal community of pig manure and chemical fertilization treatment was positively correlated with soil NO_3_-N in both two seasons, and the fungal community of rabbit manure fertilization treatment was positively correlated with soil EC, SOC, TP concentration and soil pH in the second season.

## Discussion

### Effect of organic fertilizer on yield, quality of *Brassica rapa* var. *Chinensis* and soil physico-chemical properties

The aim of this study was to investigate the effect of organic manure on *Brassica rapa* var. *Chinensis* yield and quality, soil physicochemical properties, and soil microbial community composition. For sustainable agriculture, organic fertilization is inevitable in the future as it improves soil properties, increases crop productivity and maintains crop quality (Adekiya et al., 2020). In our study, chemical fertilization significantly increased the yield of *Brassica rapa* var. *Chinensis* in the first season compared to organic fertilization (Table 2), when the total nitrogen content of organic fertilization is twice that of chemical fertilization, which means that the mineralization rate of organic fertilization is slow, and the nutrients in the soil cannot meet the needs of *Brassica rapa* var. *Chinensis*. This is because the nitrate and ammonium roots of compound fertilizers can be directly absorbed by the plants after fertilization, whereas organic fertilizers must first be mineralized into inorganic nutrients by soil microbiota before being absorbed and assimilated by plants (Alizadeh et al., 2012). The supply of nitrogen from organic fertilizers depends on their rate of mineralization, but in the first season, soil temperatures are relatively low and the rate of mineralization of organic fertilizers is also low, resulting in inadequate nutrient supply (Geisseler et al., 2021; Cannavo et al., 2022). Although the yield of *Brassica rapa* var. *Chinensis* in the chemical fertilizer treatment was significantly lower than those of organic fertilizer treatments in the second season (Table 2), this is because the fertilizer is reapplied on the basis of the first season, which increases the total nutrient content in the soil, at the same time, the rate of soil mineralization increases with the increase in temperature and the nutrients in the soil applied with organic fertilizer can continuously meet the needs of the crop in the second season(Kelderer et al., 2008; Bebber and Richards, 2022; Cannavo et al., 2022). And that the application of organic fertilizers increased the input of organic carbon and nitrogen, replenished the carbon source, promoted the reproduction of soil microorganisms, and also improved the mineralization of organic fertilizers and increased the nutrients available in the soil to meet the needs of crops (Birkhofer et al., 2008; Anggraheni et al., 2019; Wang et al., 2020a).

Organic fertilizers (pig manure and rabbit manure fertilizer) significantly increased the soluble sugar content of *Brassica rapa* var. *Chinensis*, especially in the first season, and significantly decreased the nitrate nitrogen content of *Brassica rapa* var. *Chinensis* (Table 2), and these results were consistent with the results of previous studies (Xu et al., 2002; Li et al., 2017; Youssef and Eissa, 2017; Serri et al., 2021). Consistent with the results of our study (Table 3), previous research also found that organic fertilization significantly increased soil organic carbon, total nitrogen, total phosphorus and pH (Li X. et al., 2021), and chemical fertilization decreased soil pH (Li et al., 2019), but rabbit manure fertilizer increased soil EC compared to chemical fertilization (El-Mogy et al., 2020). Rabbit manure fertilizer decreased the nitrate content of *Brassica rapa* var. *Chinensis* (Table 3). The low nitrate content was caused by the lower nitrate content of rabbit manure fertilizer in soil (Li et al., 2017) and the correlation between soil nitrate content and *Brassica rapa* var. *Chinensis* nitrate content was significant and positive (*p* = 0.876). However, the nitrate content of vegetables and soil after application of pig manure was opposite to that of rabbit manure.

### Effects of three fertilizers on diversity and abundance of the soil microbial community

Using high-throughput sequencing, three fertilization treatments were compared in two seasons of *Brassica rapa* var. *Chinensis* soil. Fertilization with organic and chemical fertilizers profoundly affected the diversity, richness, structure and activity of soil microbial communities (Lazcano et al., 2013; Francioli et al., 2016; Gu et al., 2017). Previous studies suggested that organic fertilizer increased bacterial species’ richness and diversity (Lazcano et al., 2013) and significantly increased α-diversity, such as Chao1 and Shannon index of soil bacteria (Xue et al., 2018), and our results were consistent with these findings (Table 5). This is because organic fertilizer provides sufficient substrate for soil bacteria (Li et al., 2022) and a large number of soil microorganisms (Watts et al., 2010), leading to an increase in microbial diversity. On the other hand, organic fertilizer had no significant effect on fungal species’ richness and diversity (Francioli et al., 2016; Lin et al., 2019; Qi et al., 2022), and we have similar results (Table 5). This may be due to nutrient enrichment caused by fertilization (Jiang et al., 2021), for example, fertilizer increases fungal size, which reduces fungal biodiversity and changes community composition (Zhou et al., 2016). Based on the NMDS analysis, it was found that differences in bacterial community structure were highlighted among the three fertilization treatments in two seasons (Figure 2), this result is consistent with previous study (Gu et al., 2017; Wu et al., 2020). This may be because organic fertilizer provides a greater diversity of potential substrates for bacterial growth and reproduction, and at the same time bacteria in organic fertilizer could also increase soil enhance microbial biomass (Dong et al., 2014). Previous research on the sensitivity of fungi to fertilizer has been inconsistent. Studies suggested that fungi were not significantly affected by different fertilization treatments, and possibly because conventional tillage or invasive land management (e.g., fertilization) increased bacterial activity and reduced fungal activity, whereas fungi dominated under no-tillage or less invasive land management (de Vries et al., 2006). However, other studies have suggested that fungal communities are more resilient to environmental change than bacterial communities, and that bacterial communities have broader adaptive options (Lin et al., 2019). While our study results showed that differences in fungal community structure were highlighted between the three fertilizer treatments and not between the two seasons (Figure 2), this suggests that fungal community structure is strongly influenced by fertilizer type and is not affected by time (two seasons).

### Effects of three fertilizers on the composition of the soil microbial community

In this study, the abundance of *unidentified bacteria* and *Acidobacteriota* was significantly higher in the second season than in the first season, while the abundance of *Bacteroidota* and *Crenarchaeota* was lower than in the first season (Figure 3), indicating that bacteria are more sensitive to the environment (Lin et al., 2019). *Proteobacteria* are the most abundant bacteria in the soil in our experiment*. Proteobacteria* predominate in different soil environments and are mostly Gram-negative (Liang et al., 2018), which was expected to enhance the biological cycling of essential micro- or macro- nutrients and improve soil fertility and plant growth efficiency (Lesaulnier et al., 2008). In our study, *Proteobacteria* had an absolute advantages in different treatment groups, their abundance ranged from 23 to 37%, and 6 of the top 10 bacterial genera belonged to *Proteobacteria*. Previous research suggested that the relative abundance of *Acidobacteria* was negatively correlated with soil pH (Jones et al., 2009). In our study, pig manure and rabbit manure fertilizer increased soil pH and decreased the relative abundance of soil *Acidobacteria*, compared to chemical fertilizer in the second season (Figure 3III), which was consistent with previous study results (Ma et al., 2021). Compost application has been reported to decrease the relative abundance of *Actinobacteria* (Liang et al., 2018; Ma et al., 2021), but, our study showed that rabbit manure fertilizer significantly increased the abundance of *Actinobacteria* in the second season.

Our study results showed that organic fertilizer, especially rabbit manure, reduced the relative abundance of *Ascomycota* compared to chemical fertilizer (Figure 4), this trend may be due to the more stable form of organic substances after a fermentation process during the maturation of manure (Hannula et al., 2021). Similar results were found in previous studies which showed that mineral N fertilizer promoted fungal growth and organic fertilizer reduced fungal growth (Wang et al., 2017; Hannula et al., 2021). *Basidiomycota* are widely regarded as lignin decomposers and are therefore important for soil carbon cycling (Hanson et al., 2008). Our results showed that organic fertilizer reduced the abundance of soil *Basidiomycota*, as the beneficial function of *Basidiomycota* could be affected by high soil N level (Paungfoo-Lonhienne et al., 2015) and perhaps also by the inhibition of rabbit manure fertilizer.

### Relationships between microbial communities and soil properties

According to Xue et al. (2018), soil properties, including nutrients (e.g., total C, total N, P,EC), are more correlated with the absolute abundance of microbes, and EC, clay content and pH accounted of the variation in soil microbial structure in southeast Australia. It has been reported that organic fertilizers can influence the structure of bacterial communities by altering soil properties in a soil type (Li P. et al., 2021; Iqbal et al., 2022). Wu et al. reported that the bacterial community was influenced by soil EC and soil carbon, while the fungal community was more influenced by alkaline nitrogen (Wu et al., 2020). Our study also found that pig manure fertilizer and rabbit manure fertilizer altered soil physicochemical properties (Table 3). Distance-based redundancy analysis (dbRDA) according to the relative abundance of OTUs further showed that, overall, soil EC, TN, and SOC concentration were the most important physicochemical factors to assemble the bacterial community structure in *Brassica rapa* var. *Chinensis* soil, and we also found that in the second season, soil EC and NO_3_-N content had the great effect on the soil bacterial community structure of pig manure fertilizer application and soil TN of rabbit manure fertilizer application (Figure 5I). dbRDA results showed that soil NO_3_-N, EC, SOC concentration and soil pH were fungal community structure in *Brassica rapa* var. *Chinensis* soil, and we also found that soil EC, SOC, pH had the great effect on the soil fungal community structure of rabbit manure fertilizer application and soil NO_3_-N of chemical fertilizer and pig manure fertilizer treatment soil (Figure 5II).

## Conclusion

(1) With the increase of time, the advantage of using organic fertilizer to increase the yield of *Brassica rapa* var. *Chinensis* in the second season gradually emerged. The rabbit manure fertilizer significantly (*p* ≤ 5%) reduced the NO_3_-N content of fresh *Brassica rapa* var. *Chinensis*.

(2) The organic fertilizer increased the concentration of total nitrogen, total phosphorus and organic carbon in soil and the rabbit manure fertilizer increased soil pH and EC and significantly (*p* ≤ 5%) reduced soil NO_3_-N content.

(3) Organic fertilizer significantly (*p* ≤ 5%) improved the diversity and richness of soil bacteria in *Brassica rapa* var. *Chinensis* soil, but had no significant effect on soil fungi. There were significant differences (*p* ≤ 5%) in the bacterial community structures between different treatments in two seasons, and significant differences (*p* ≤ 5%) in the fungal community structures between fertilizer treatments, but not between two seasons.

(4) Soil EC, TN and organic carbon content were the most important physicochemical factors in determining the bacterial community structure in *Brassica rapa* var. *Chinensis* soils, and soil NO_3_-N, EC, SOC concentration and soil pH in the fungal community structure.

## Data availability statement

The raw sequence data reported in this study have been deposited in the Genome Sequence Archive (Genomics, Proteomics & Bioinformatics 2021) in National Genomics Data Center (Nucleic Acids Res 2022), China National Center for Bioinformation/Beijing Institute of Genomics, Chinese Academy of Sciences (https://ngdc.cncb.ac.cn/gsa), under the accession number CRA011178.

## Author contributions

XZ, HG, and JY: conceptualization. XZ, JY, and XP: methodology. JL and PZ: software. LS and JY: validation. XZ, FQ, and XP: formal analysis. JY: investigation. GH: resources. XZ and JY: data curation. XZ: writing—original draft preparation, writing—review and editing, and project administration. FQ: visualization. JL: supervision. JY and XP: funding acquisition. All authors contributed to the article and approved the submitted version.

## Funding

This research was funded by the the earmarked fund for China Agriculture Research System (grant number: CAR-43-G-2), the Jiangsu Characteristic Livestock and Poultry Industry Technology System (grant number: JATS[2022]244), Jiangsu Province Vice President of Science and Technology Project (grant number: FZ20220074), the Jiangsu Industry University Research Cooperation Project (590) and the One Zone, One Center Joint Special Project (grant number: 202212005).

## Conflict of interest

The authors declare that the research was conducted in the absence of any commercial or financial relationships that could be construed as a potential conflict of interest.

## Publisher’s note

All claims expressed in this article are solely those of the authors and do not necessarily represent those of their affiliated organizations, or those of the publisher, the editors and the reviewers. Any product that may be evaluated in this article, or claim that may be made by its manufacturer, is not guaranteed or endorsed by the publisher.
